# Dietary Behaviors among New Users of Meal-Kit Services during the Early Months of the COVID-19 Pandemic

**DOI:** 10.3390/nu14193953

**Published:** 2022-09-23

**Authors:** Joelle N. Robinson-Oghogho, Roland J. Thorpe, Roni A. Neff

**Affiliations:** 1 Department of Health Behavior and Society, Bloomberg School of Public Health, Johns Hopkins University, Baltimore, MD 21205, USA; 2 Hopkins Center for Health Disparities Solutions, Bloomberg School of Public Health, Johns Hopkins University, Baltimore, MD 21205, USA; 3 Department of Environmental Health and Engineering, Bloomberg School of Public Health, Johns Hopkins University, Baltimore, MD 21205, USA; 4 Johns Hopkins Center for a Livable Future, Bloomberg School of Public Health, Johns Hopkins University, Baltimore, MD 21202, USA

**Keywords:** meal-kits, COVID-19, dietary changes, eating behaviors

## Abstract

The COVID-19 pandemic changed the way people acquired food, including increased use of meal-kit delivery services. Investigators analyzed data from a national survey of US adults collected between July 2020 and September 2020, to describe new users of meal-kit services during the pandemic and explore associations between new use of meal-kits and dietary behaviors. Bivariate and multivariate regression analyses were conducted to identify differences in demographic characteristics and reported dietary behaviors between new and never meal-kit users. Nearly all new meal-kit users were under the age of 55 years (92.5%), lived in urban areas (90.1%), and reported having children in their households (82%). A higher proportion of new users were current SNAP participants (32.8%) compared to never users (17.1%). Compared to never users, new users of meal-kit services reported eating more fruits and vegetables (PR: 1.95, 95% CI: 1.42, 2.68), and more red and processed meats (PR: 2.39, 95% CI: 1.49–3.85) since the pandemic began. Results suggest that meal-kit services may have been a useful resource for certain populations during the early months of COVID-19 and are potentially associated with increased consumption of certain foods. Further research examining the continued use and the influence of meal-kit services on diet is needed.

## 1. Introduction

On 11 March 2020, the World Health Organization announced COVID-19 as a global pandemic. Attempts to prevent the transmission of the virus via stay-at-home orders caused many people to remain sequestered in their homes [1,2]. Mandated or self-imposed practices such as limits on the number of people allowed in stores, changes in store hours, or reductions in the frequency of trips to food retail locations shifted how and where people acquired food. For example, use of food delivery services, namely grocery and meal-kit delivery services, increased during the COVID-19 pandemic [3,4,5]. 

In addition to avoiding in-store shopping, US consumers also reported cooking more since the pandemic [6,7]. These behavior changes are important in the context of nutritional outcomes because cooking has repeatedly been found to be associated with improved diets [8,9,10]. Meals prepared at home have been shown to be more nutritious and more likely to contain vegetables than foods purchased at restaurants or other prepared food vendors [7,11]. While both grocery delivery and meal-kit services provide customers with the ability to avoid in-store shopping and have the potential to increase home cooking [12], meal-kits may present a unique opportunity to improve dietary outcomes by decreasing some of the additional barriers to cooking such as challenges with meal planning, shopping, and cooking skills. Meal-kits are typically subscription services that provide customers with a weekly delivery of pre-portioned food ingredients and recipes. Depending on the service, two to six meals are provided each week and customers have the option to choose their meals and select how many servings each meal provides based on their household’s needs (i.e., two servings per meal vs. four servings per meal).

Although some research has been done to explore the utility and acceptability of meal-kits in different populations [13,14], few studies have examined the health promoting capabilities of meal-kit services. One study conducted a nutritional analysis of the meals offered by a popular international meal-kit company (Hello Fresh) in Australia between 2017 and 2018. Researchers found that the meals frequently included vegetable ingredients but that many meals also contained high levels of sodium [15]. Given that some meal-kit services specifically market their meals as healthy [16,17], and the increased use of meal-kit services that have been reported since the pandemic, it would be worthwhile to further investigate how meal-kits might function as a tool for promoting improved health behaviors. 

Historically, users of meal-kit services have been characterized as younger adults (ages 25–44) with higher household incomes and higher levels of educational attainment compared to the overall population [18,19]. However, it is possible that the COVID-19 pandemic fostered the use of these services among a broader population, due to factors such as widespread restaurant closures and broad concerns about virus transmission through in-person food shopping experiences. Understanding who is now using these services and the potential influence of their use on dietary behaviors may shed light on an important opportunity to improve nutrition during public health emergencies, and generally. Using data from a US national study examining the impact of the COVID-19 pandemic on food access, perceptions, and behaviors this study sought to identify the characteristics of people who are newly using meal-kit services during the first six months of the pandemic. To further understand the potential influence of meal-kit subscriptions on nutritional outcomes we also explore if there are differences in the reported dietary behaviors between new users of these services and never users. 

## 2. Materials and Methods

### 2.1. Data

Data come from the first wave of the National Food Access and COVID Research Survey [20]. In this survey, a sample of 1510 adult US residents was recruited using panels administered by the Qualtrics survey research firm [21]. The survey was administered online from July 2020 through September 2020. Quota sampling was used to recruit respondents who were representative of the US population by race. Respondents from lower-income households (i.e., with an annual income in 2019 lower than USD 50,000) were oversampled. Participants were asked questions about food access and security, food behaviors, and demographic characteristics for periods “prior to the pandemic” (i.e., from March 2019 to 11 March 2020) and “since the pandemic” (i.e., from 11 March 2020, to the time of the survey). Our analytic sample included 1413 participants who responded to the survey question about the use of meal-kit delivery services—our main independent variable. 

### 2.2. Measures

#### 2.2.1. Dietary Behaviors

Reported changes in dietary behaviors are the main outcome variable of interest in this analysis. Several questions were asked to assess changes in dietary behaviors since the COVID-19 pandemic. To assess changes in fruit and vegetable consumption participants were asked “Compared to before the COVID-19 outbreak, how have you been eating in the past month during the COVID-19 outbreak (since 11 March).” Participants indicated if they thought they had been eating more, less, or about the same amount of fruits and vegetables per day, and if they thought they had been eating more, less, or about the same amount of processed and red meats. For our analysis, we grouped these responses into a binary variable to categorize reported changes in dietary behavior as “eating more” or “eating less or the same” since the pandemic.

Two separate questions were used to quantify the fruits and vegetables participants were consuming during the pandemic. Participants were asked, in the past month, “About how many cups of vegetables (including 100% vegetable juice) do you eat or drink each day? Examples of 1 cup of vegetables include: 1 cup of cooked leafy greens, 2 cups of lettuce or raw greens, 12 baby carrots, 1 medium potato, or 1 large raw tomato.” They were also asked, “About how many cups of fruit (including 100% pure fruit juice) do you eat or drink each day? Examples of 1 cup of fruit include: 1 small apple, 1 large banana, 1 cup (8 oz.) of 100% juice or canned fruit, or ½ cup of dried fruit.” Response options to these questions were “none” “½cup or less” “½–1 cup,” “1–2 cups,” “2–3 cups,” “3–4 cups,” and “4 cups or more.” For our analysis, these response options were collapsed into the following five categories: none, ½ cup or less, ½–1 cup, 1–2 cups, and 2 or more cups. These questions have been found to be valid measures to broadly screen for fruit and vegetable intake [22]. 

Additionally, two separate questions taken from the NHANES 2009–2010 Dietary Screener Questionnaire [23] were used to quantify the red and processed meats participants were consuming during the pandemic. Participants were asked in the past month, “How often did you eat red meat (such as beef, pork, ham, sausage, veal lamb)? Do not include chicken, turkey or seafood. Include red meat you had in sandwiches, lasagna, stew, and other mixtures.” and “How often did you eat any processed meat, such as bacon, lunch meats, or hot dogs? Include processed meats you had in sandwiches, soups, pizza, casseroles, and other mixtures. Processed meats are those preserved by smoking, curing, or salting, or by the addition of preservatives.” Response options for these questions were “never,” “1 time last month” “2–3 times last month,” “1 time per week,” “3–4 times per week,” “5–6 times per week,” “1 time per day,” and “2 or more times per day.” For our analysis, these response options were collapsed into the following four categories: Never—3 times last month, 1–2 times per week, 3–6 times per week, and 1 or more times per day. 

#### 2.2.2. Use of Meal-Kit Services

The use of meal-kit services is our main independent variable of interest. To determine places where consumers acquired food, survey participants were asked to select from a list all the places their household used to get food in the year before the COVID-19 outbreak, and since the COVID-19 outbreak (i.e., 11 March 2020). Our analyses focus on participants who indicated that they used meal-kit delivery services such as Blue Apron. Participants could indicate that they used the service in the year before COVID-19, since COVID-19, or at both time periods. Survey participants were categorized as new users of meal-kits if they indicated they used meal-kit delivery services since the pandemic but not before the pandemic. Participants who indicated the use of meal-kits before or at both time periods were categorized as prior users. Participants who did not indicate the use of meal-kit services during either time period were categorized as never users. 

#### 2.2.3. Demographic Characteristics

To characterize meal-kit users, we examined nine demographic variables: household income, educational attainment, age, race, household composition, urbanicity, food security status, Supplemental Nutrition Assistance Program (SNAP) participation, and quarantine history. Annual household income was grouped into four categories: less than USD 25,000, USD 25,000-USD 49,999, USD 50,000-USD 99,999, USD 100,000 or more. Educational attainment was categorized as high school or less, some college or associate degree, college degree or advanced degree. Participants’ age was grouped into three categories: 18–34, 35–54, and 55 years and older. Participants’ race was categorized as White; Black or African American; Asian or Pacific Islander; and Native American, Multiple Races, or Other. Household composition was categorized as households with children ages 17 years or younger and those without any children. Urban status was defined as a participant’s zip code having 50% or more of its census tracts categorized as urban (i.e., tracts with more than 2500 people) by the USDA Food Atlas database [24,25] and were identified using the U.S. Department of Housing and Urban Development (HUD) and United States Postal Service (USPS) zip code crosswalk files [26]. Food security status was measured using the USDA’s six-item household food security survey module [27] for the year before COVID-19 and since COVID-19. Participants who affirmatively answered two or more questions in the module were categorized as food insecure. Food security was then categorized into three groups; (1) food-secure, defined as those who were food secure at the time of the survey, (2) persistently food insecure, defined as those who experienced food insecurity in the year before the pandemic and since the pandemic began, and (3) newly food insecure, defined as those who were food secure in the year before the pandemic but experienced food insecurity since the pandemic began. SNAP participation was assessed using the question, “Which of the following food assistance programs did your household use in the year before the COVID-19 outbreak, if any, and since the COVID-19 outbreak (March 11)?” Respondents who indicated that their household used “SNAP or Food Stamps (including pandemic-EBT or P-EBT)” since the pandemic were categorized as current SNAP participants. Quarantine history was assessed using the question “Have you had to quarantine in your home due to COVID-19, for example because of illness, exposure, or symptoms?” with Yes or No response options.

### 2.3. Analysis

We conducted descriptive analyses to examine the demographic characteristics and reported dietary behaviors of new and never users of meal-kit delivery services. Chi-squared tests were used to assess if there were significant differences in the proportional distribution of demographic characteristics and reported dietary behaviors between new and never users. 

To assess associations between new use of meal-kit services and reported changes in dietary behaviors since the pandemic (i.e., participants reporting eating more vs. less or the same amount of fruits and vegetables; red and processed meats), modified Poisson regressions were used. Modified Poisson regressions have been used to provide estimates of prevalence ratios for binary outcomes in cross-sectional studies when the outcome is not rare (i.e., greater than 10%) [28,29]. 

To assess associations between new use of meal-kit services and the quantity of foods consumed during the pandemic, ordinal logistic regression models were specified to determine if there were significant differences in the proportional odds of reporting an increased level of consumption for each food type (i.e., fruits, vegetables, red meats, processed meats) between new users and never users of meal-kit services. Before executing the ordinal regressions, we tested and confirmed that the proportional hazard assumption was met for each outcome variable assessed using the ordinal regression models [30,31]. 

For each outcome variable, we conducted both unadjusted and adjusted analyses. Adjusted models control for household income, educational attainment, age group, race, urbanicity, quarantine history, food security status, and SNAP participation. All analyses were weighted to be representative of the US population by race and income. All analyses were conducted using STATA 15.1 [32]. *p*-values less than 0.05 were considered statistically significant.

## 3. Results

Table 1 compares new users of meal-kit services to those who reported never using meal-kit services. Many new users of meal kit services lived in households with higher pre-pandemic incomes. Fifty percent of new users reported household incomes of USD 100,000 or greater in 2019. A majority (67%) of new users had high educational attainment, reporting earning college or advanced degrees. Nearly all new users were under the age of 55 years (92.5%), lived in urban areas (90.1%), and reported having children in their households (82%). Sixty-eight percent of new users reported experiencing either new or persistent food insecurity. A higher proportion of new users were current SNAP participants (32.8%) compared to never users (17.1%). Additionally, 34.7% of new users of meal-kit services reported having had to quarantine at home due to illness or exposure to COVID-19. 

Reported dietary behaviors of new and never users of meal-kit services are displayed in Table 2. During the first six months of the pandemic, 46% of new meal-kit users reported eating more fruits and vegetables since the pandemic began, while 16.4% of never users reported consuming more fruits and vegetables since the pandemic. Among new users of meal-kit services, 24.5% reported eating or drinking two or more cups of vegetables each day, whereas 17.9% of never users reported eating or drinking two or more cups of vegetables each day. More than 1 in 5 new users of meal-kit services reported eating more red and processed meats since the pandemic (23.9%), compared to only 8.6% of never users of meal-kit services (Table 2). Bivariate associations between the use of meal-kit services and dietary behaviors indicate that there were differences between new and never users of meal-kit services on reported changes in fruit and vegetable consumption and red and processed meat consumption (Table 2). 

Table 3 displays associations between new use of meal-kit services and reported changes in dietary behaviors after controlling for household income, educational attainment, age, race, urbanicity, quarantine history, food security status, and SNAP participation. New users of meal-kit services had nearly twice the prevalence of reporting that they ate more fruits and vegetables since the pandemic began than never users (PR: 1.95, 95% CI: 1.42, 2.68). New users of meal-kit services also reported eating more red and processed meats since the pandemic began at a prevalence 2.39 times (95% CI:1.49–3.85) higher than that of never users. 

Associations between new use of meal-kit services and the reported quantity consumed of specific foods during the first six months of the pandemic are reported in Table 4. Adjusted analyses indicate that compared to never users, new users of meal-kit services had twice the proportional odds (OR: 1.99, 95% CI: 1.32–2.97) of consuming two or more cups of fruits vs. anything less than two or more cups. New users of meal-kit services also had higher proportional odds of consuming processed meats more frequently than never users during the pandemic (OR: 1.61, 95% CI: 1.07–2.43). There were no statistically significant differences in the proportional odds of consuming an increased level of vegetables or red meat between new users and never users.

## 4. Discussion

The objective of this analysis was to characterize new users of meal-kit services and explore associations between the use of meal-kit services and reported dietary behaviors during the COVID-19 pandemic. Based on data collected during the first six months of the COVID 19 pandemic our findings indicate that the majority of new users of meal-kit services had pre-pandemic incomes of >USD 100,000, advanced educational attainment (i.e., college or advanced degrees), were <55 years old, and had children in the household. Regarding the associations between the use of meal-kit services and dietary behaviors, our findings indicated that compared to never users, a greater proportion of new meal-kit users perceived themselves to generally be eating more fruits and vegetables and more red and processed meat during the pandemic than in the previous year. Additionally, our findings indicated that new users of meal-kit services had greater proportional odds of reporting consuming higher quantities of fruits and eating processed meats more frequently during the pandemic, than never users. These findings provide mixed evidence on the utility of meal-kits for improving dietary behaviors, but overall suggest that using meal-kit services may be associated with higher consumption of both foods considered nutritious (i.e., fruits) as well as less nutritious (i.e., processed meats) [33,34].

Our characterization of users aligns with what has been previously reported about users of meal-kit services [35]. Additionally, we compared the demographic characteristics of those who reported using meal-kit services prior to the pandemic to those who were new users. With the exception of educational attainment, there were no statistically significant differences between the two groups, suggesting that the early months of the pandemic did not result in totally new populations using these services. Although our characterization of meal-kit users aligns with previous descriptions, it is worth noting that among new users of meal-kit services 52% reported persistent food insecurity, 33% currently received SNAP or Pandemic EBT benefits, and 26% were from households that could be considered low- and middle-income (i.e., making less than USD 50,000). Although the majority of new meal-kit users had pre-pandemic incomes of USD 100,000 or more, other analyses using these survey data indicate that many of the people in these higher-income households also reported experiencing job disruptions and subsequent food insecurity during the pandemic [36]. This suggests that during times of emergency people with historically high incomes may still experience disruptions that make them vulnerable to food insecurity, and that people in households with fewer expendable resources for food, who are not traditionally described as the population using meal-kits, may still be an important segment to consider. Although the SNAP online grocery purchasing pilot program had expanded to 47 states by the end of 2020 [37], it is unlikely that the participants in this study used their benefits to purchase meal-kit services since few, if any, meal-kit companies were accepting SNAP for online payments in the early months of the pandemic [38]. However, if a retailer is able to meet the online purchasing requirements established by the USDA, meal-kits would be SNAP eligible.

Further research exploring if older adults or people in households characterized as low or middle income perceive meal-kit services as being a viable food acquisition option, could help illuminate if meal-kits services might offer additional benefits, or alleviate challenges experienced in these populations. For example, understanding how the cost of meal-kit services compares to grocery delivery or restaurant meals, or the extent meal-kits save time and effort compared to conventional cooking would complement further assessments of the potential benefits of meal-kits for encouraging home cooking. Additional research on the influence of meal-kits on dietary behaviors is also warranted as our findings provided mixed indications about how and if the use of meal-kits impacts diet. It is possible that people with existing motivations for healthy eating or interests in cooking may be more inclined to use meal-kit services. Longitudinal study designs that use more precise dietary assessment measures than those used in this analysis, and that account for values and perceptions related to cooking and healthy eating behaviors before utilization of meal-kit services, will be better suited to assess the influence of meal-kit services on diets. Additionally, as other non-delivery meal-kit options become available, such as meal-kit packages sold in grocery stores, differentiating between the types of meal-kit options will also be an important consideration for researchers. 

As suggested above, our analysis has several limitations. First, the dataset used in this analysis did not include any information about the composition or level of utilization of the meals received through meal-kit services. As this was a secondary data analysis, we were limited to the existing survey questions. Knowing the types of food consumers received, how much, or how often they utilized meal-kit services would provide a better understanding of the relationship between use and diet. As meal-kits typically do not contain fruit items, our findings that new users consumed greater amounts of fruits suggest that survey participants may have included other services in their reporting on meal-kits, or that the associations we found between the use of meal-kits and reported dietary behaviors are indirectly related. Since survey participants sourced food from other locations in addition to meal-kits it is possible that people using meal-kits may generally purchase and consume healthier foods than never users of meal-kits. Further, the questionnaire used to assess fruit and vegetable intake is known to overestimate fruit consumption so our results may also reflect this issue [22]. Finally, since some of the questions used in our analyses asked survey participants to compare their eating behaviors at the time of the survey to the time before the pandemic, it is possible that people who experienced food-related challenges since the pandemic may remember their eating habits more, or less, accurately than those who did not experience challenges, thereby creating potential recall bias.

Nonetheless, as few studies [39,40] have been published in the peer-reviewed literature assessing the associations between meal-kit use and nutritional outcomes, this analysis presents foundational estimates that future research can build on. Data from this analysis come from a nationally representative survey collected using online panels which have been shown to be a valid method for participant recruitment [21]. Additionally, data from this analysis were collected between July and September 2020; representing a stage of the COVID-19 pandemic when consumers were likely to have made several adjustments to their food-related behaviors. Information from this time period is particularly valuable as it provides insights into food-related behaviors during a public health emergency. This study thereby adds to the limited literature related to the influence of meal-kit utilization on dietary behaviors in the context of COVID-19. As we are likely to face future public health emergencies and as we continue to explore ways to improve health and dietary outcomes generally, further studies are warranted to specifically examine the role of meal-kit services as a potential public health promotion tool, both in disasters and everyday times.

## 5. Conclusions

During the COVID-19 pandemic, consumers’ use of meal-kit services increased [4,41]. The results from our analysis suggest that meal-kits were a useful service for people who had to quarantine at home during the pandemic and that people with lower incomes and those who may have experienced food insecurity are segments of the population using meal-kits that have been previously overlooked. Further, our results suggest that those using meal-kit services reported greater consumption of certain types of foods compared to those who never used meal-kit services. While meal-kits seem to have offered an opportunity for consumers to avoid in-person food shopping during COVID-19, similar to grocery delivery services [42], it is possible that meal-kit delivery services could also be a method to increase food access generally, specifically for those living in areas that have low access to healthy food options. Meal-kits may also offer the additional benefit of reducing the time required to cook if meal ingredients are already pre-portioned or chopped. In 2021 vaccines against COVID-19 became widely available and restrictions related to the pandemic started to recede [2,43]. Moving forward it will be important to explore the extent to which utilization of meal-kit services remains stable, and if so, to explore their influence on diet more thoroughly. 

## Figures and Tables

**Table 1:** Weighted Distribution of Characteristics of Participants in the National Food Access and COVID Research Survey, by Meal-Kit Delivery Service Use During the Early Months of COVID-19 Pandemic.

	Use of Meal-Kit Services		
	Prior User *n* = 161	New User *n* = 148	Never User *n* = 1104
**Total, %**	14.0	12.1	74.0
**Household Income, % ^b^**			
<USD 25,000	8.9	11.8	23.6
USD 25,000-USD 49,999	10.4	14.1	25.4
USD 50,000-USD 99,000	27.7	23.4	31.2
≥USD 100,000	53.0	50.7	19.9
**Education, % ^a,b^**			
High School or Less	7.7	13.3	19.6
Some College/Associate	13.1	19.7	31.0
College/Advanced	79.1	67.0	49.4
**Age, % ^b^**			
18–34	42.1	41.8	27.3
35–54	54.4	50.7	31.8
55+	3.5	7.5	40.9
**Households with Children, % ^b^**			
Yes	79.3	82.1	26.7
**Race, %**			
White	85.7	80.0	75.4
Black	8.02	10.0	12.0
Asian, Pacific Islander	2.6	4.6	4.9
Native Am., Multi, Other	3.7	5.4	7.7
**Urbanicity, % ^b^**			
Urban	90.6	90.1	82.6
Non-Urban	9.4	9.9	17.4
**Food Security Status, % ^b^**			
Food Secure Now	41.7	32.3	77.0
Newly Insecure	12.6	15.5	7.8
Persistently Insecure	45.7	52.2	15.2
**SNAP Participant, % ^b^**			
Yes	24.0	32.8	17.1
**Had to Quarantine, % ^b^**			
Yes	36.9	34.7	13.5

^a^ chi-square test indicates a significant difference in the distribution of the respective demographic characteristic between prior users and new users of meal-kits at a 95% confidence level. ^b^ chi-square test indicates a significant difference in the distribution of the respective demographic characteristics between new users and never users of meal-kits at a 95% confidence level. Prior Users = use of meal-kit services before the pandemic, or before and during the pandemic New Users = use of meal-kit services during the pandemic, but not before the pandemic. Never Users = no use of meal-kit services either before or during the pandemic.

**Table 2:** Weighted Distribution of Reported Dietary Behaviors Among Never Users and New Users of Meal-Kit Delivery Services During the Early Months of COVID-19 Pandemic: Results from the National Food Access and COVID Research Survey.

	Meal-Kit Delivery Services		
	New User *n* = 148	Never User *n* = 1104	*p*-Value
**Fruits and Veg Consumption Compared to Before C-19, %**			**<0.000**
Eating the Same or Less	54.0	83.6	
Eating More	46.0	16.4	
**Red and Processed Meat Consumption Compared to Before C-19, %**			**<0.000**
Eating the Same or Less	76.1	91.7	
Eating More	23.9	8.3	
**Quantity of Fruit Consumption, %**			**0.003**
None	6.7	15.1	
½ cup or less	15.3	19.6	
½–1 cup	22.0	22.5	
1–2 cups	30.0	27.9	
2 or more cups	26.0	15.0	
**Quantity of Vegetable Consumption, %**			0.095
None	9.8	9.5	
½ cup or less	9.7	18.1	
½–1 cup	23.1	23.7	
1–2 cups	32.9	30.8	
2 or more cups	24.5	17.9	
**Frequency of Red Meat Consumption, %**			0.331
Never—3 times last month	27.6	32.3	
1–2 times per week	34.2	33.1	
3–6 times per week	33.2	27.1	
1 or more times per day	4.9	7.5	
**Frequency of Processed Meat Consumption, %**			**0.022**
Never—3 times last month	33.1	43.6	
1–2 times per week	34.6	35.3	
3–6 times per week	27.6	17.8	
1 or more times per day	4.8	3.3	

New User = use of meal-kit services during the pandemic, but not before the pandemic. Never Users = no use of meal-kit services either before or during the pandemic. Bolded values indicate statistically significant difference at a 95% confidence interval.

**Table 3:** Associations of Reported Changes in Dietary Behaviors and New Use of Meal Kit Delivery Services during the Early Months of COVID-19 Pandemic: Results from the National Food Access and COVID Research Survey.

Reported Change in Consumption Since Pandemic Prevalence Ratio (95% Confidence Interval)				
	Eating More Fruits and Veg.		Eating More Red and Processed Meat	
	vs. Less or Same		vs. Less or Same	
	Unadjusted Model	Adjusted Model *	Unadjusted Model	Adjusted Model *
Meal Kit Service:	**PR: 2.81**	**PR: 1.95**	**PR: 2.88**	**PR: 2.39**
New Use vs. Never Use (Ref)	**(2.21–3.56)**	**(1.42–2.68)**	**(1.99–4.17)**	**(1.49–3.85)**

* Adjusted models control for household income, educational attainment, age group, race, urbanicity, quarantine history, food security status, and SNAP participation. Ref = reference group. Bolded values indicate statistically significant difference at a 95% confidence interval.

**Table 4:** Associations of Reported Quantity of Foods Consumed and New Use of Meal Kit Delivery Services during the Early Months of COVID-19 Pandemic: Results from the National Food Access and COVID Research Survey.

Reported Consumption Quantity during the Pandemic Proportional Odds Ratio (95% Confidence Interval)								
	Higher Quantity of Fruit Consumption		Higher Quantity of Vegetable Consumption		Higher Frequency of Red Meat Consumption		Higher Frequency of Processed Meat Consumption	
	Unadjusted Model	Adjusted Model *	Unadjusted Model	Adjusted Mode *	Unadjusted Model	Adjusted Model *	Unadjusted Model	Adjusted Model *
Meal Kit Service: New Use vs. Never Use (Ref)	**OR: 1.87 (1.35–2.58)**	**OR: 1.99** **(1.32–2.97)**	**OR: 1.44** **(1.03–2.02)**	OR: 1.50 (0.96–2.35)	OR: 1.14 (0.84–1.56)	OR: 1.40 (0.97–2.02)	**OR: 1.66** **(1.18–2.34)**	**OR: 1.61** **(1.07–2.43)**

* Adjusted models control for household income, educational attainment, age group, race, urbanicity, quarantine history, food security status, and SNAP participation. Ref = reference group. Bolded values indicate statistically significant difference at a 95% confidence interval.

## Data Availability

The survey data that support the findings of this study are available from the corresponding author, J.N.R.-O., upon reasonable request.

## References

[1] Bump P. (2020). Mapping Where America Has Been Shut Down, in The Washington Post. https://www.washingtonpost.com/politics/2020/03/17/mapping-where-america-has-been-shut-down/.

[2] (2020). Kaiser Family Foundation, Social Distancing Order Effective and Rollback Dates. https://github.com/KFFData/COVID-19-Data/tree/kff_master/State%20Policy%20Actions/State%20Social%20Distancing%20Actions.

[3] Chenarides L., Grebitus C., Lusk J.L., Printezis I. (2021). Food consumption behavior during the COVID-19 pandemic. Agribusiness.

[4] Lee S., Ham S. (2021). Food service industry in the era of COVID-19: Trends and research implications. Nutr. Res. Pr..

[5] Gelder K.V. (2022). Meal Preparation Kits Shopping Frequency in the U.S. 2019–2021. https://www.statista.com/statistics/1281661/meal-preparation-kit-order-frequency-us/#statisticContainer.

[6] Bender K.E., Badiger A., Roe B.E., Shu Y., Qi D. (2021). Consumer behavior during the COVID-19 pandemic: An analysis of food purchasing and management behaviors in U.S. households through the lens of food system resilience. Socio-Econ. Plan. Sci..

[7] Todd J.E., Mancino L., Lin B.-H. (2010). The Impact of Food Away from Home on Adult Diet Quality.

[8] Fertig A.R., Loth K., Trofholz A.C., Tate A., Miner M.H., Neumark-Sztainer D., Berge J. (2019). Compared to Pre-prepared Meals, Fully and Partly Home-Cooked Meals in Diverse Families with Young Children Are More Likely to Include Nutritious Ingredients. J. Acad. Nutr. Diet..

[9] Wolfson J.A., Bleich S.N. (2015). Is cooking at home associated with better diet quality or weight-loss intention?. Public Health Nutr..

[10] Mills S., Brown H., Wrieden W., White M., Adams J. (2017). Frequency of eating home cooked meals and potential benefits for diet and health: Cross-sectional analysis of a population-based cohort study. Int. J. Behav. Nutr. Phys. Act..

[11] Mancino L., Guthrie J., Ploeg M.V., Lin B.H. (2018). Nutritional Quality of Foods Acquired by Americans: Findings From USDA’s National Household Food Acquisition and Purchase Survey. Econ. Inf. Bull..

[12] Gelder K.V. (2022). Meal Kits in the U.S.-Statistics & Facts. https://www.statista.com/topics/3336/online-meal-kit-delivery-services-in-the-us/#topicHeader__wrapper.

[13] Oberle M.M., Loth K.A., Schendel A., Fox C.K., Gross A.C. (2020). Acceptance of a meal kit programme in an outpatient paediatric weight management clinic: A qualitative pilot study. Clin. Obes..

[14] Carman K., Sweeney L.H., House L.A., Mathews A.E., Shelnutt K.P. (2021). Acceptability and Willingness to Pay for a Meal Kit Program for African American Families with Low Income: A Pilot Study. Nutrients.

[15] Moores C.J., Bell L.K., Buckingham M.J., Dickinson K.M. (2021). Are meal kits health promoting? Nutritional analysis of meals from an Australian meal kit service. Heal. Promot. Int..

[16] Green Chef (2022). The #1 Meal Kit for Eating Well. https://www.greenchef.com.

[17] Sunbasket Healthy Meal Service for Any Lifestyle. https://sunbasket.com/menu.

[18] Leonhardt M. (2017). These 2 Charts Show Just How Popular Meal-Kit Services Are. http://money.com/money/4855511/who-buys-meal-kit-services/.

[19] Schoenbauer A. Meal Kit Market: The Whos and Whys Behind Meal Kit Buys, in Numerator. 2019. Numerator. https://www.numerator.com/resources/blog/whos-and-whys-behind-meal-kit-buys.

[20] Niles M.T., Belarmino E.H., Bertmann F., Biehl E., Neff R. (2020). Food insecurity during COVID-19: A multi-state research collaborative. medRxiv.

[21] Miller C.A., Guidry J.P., Dahman B., Thomson M.D. (2020). A Tale of Two Diverse Qualtrics Samples: Information for Online Survey Researchers. Cancer Epidemiol. Biomark. Prev..

[22] Yaroch A.L., Tooze J., Thompson F.E., Blanck H.M., Thompson O.M., Colón-Ramos U., Shaikh A., McNutt S., Nebeling L.C. (2012). Evaluation of Three Short Dietary Instruments to Assess Fruit and Vegetable Intake: The National Cancer Institute’s Food Attitudes and Behaviors Survey. J. Acad. Nutr. Diet..

[23] National Cancer Institute Dietary Screener Questionnaires (DSQ) in the NHANES 2009-10: DSQ. 14 December 2021. https://epi.grants.cancer.gov/nhanes/dietscreen/questionnaires.html.

[24] United States Department of Agriculture. Economic Research Service (2018). USDA -Food Access: DataDownload2015.xlsx. Inter-university Consortium for Political and Social Research: Ann Arbor, MI, USA. https://www.datalumos.org/datalumos/project/101441/version/V1/view?type=file&path=/datalumos/101441/fcr:versions/V1/DataDownload2015.xlsx.

[25] United States Department of Agriculture. Economic Research Service (2018). USDA-Food Access: Documentation2015.pdf. In-ter-university Consortium for Political and Social Research: Ann Arbor, MI, USA. https://www.datalumos.org/datalumos/project/101441/version/V1/view?path=/datalumos/101441/fcr:versions/V1/Documentation2015.pdf&type=file.

[26] United States Department of Housing and Urban Development (2020). HUD USPS Zip Code Crosswalk Files. ZIP-TRACT 4th Quarter 2020 Datafile. https://www.huduser.gov/portal/datasets/usps_crosswalk.htm.#data.

[27] United States Department of Agriculture Economic Research Service (2012). U.S. Household Food Security Survey Module: Six-Item Short Form. https://www.ers.usda.gov/media/8282/short2012.pdf.

[28] Zou G. (2004). A Modified Poisson Regression Approach to Prospective Studies with Binary Data. Am. J. Epidemiol..

[29] Martinez B.A.F., Leotti V.B., Silva G.D.S.E., Nunes L.N., Machado G., Corbellini L.G. (2017). Odds Ratio or Prevalence Ratio? An Overview of Reported Statistical Methods and Appropriateness of Interpretations in Cross-sectional Studies with Dichotomous Outcomes in Veterinary Medicine. Front. Veter Sci..

[30] Harrell F.E., Harrell J.F.E. (2015). Ordinal Logistic Regression, in Regression Modeling Strategies: With Applications to Linear Models, Logistic and Ordinal Regression, and Survival Analysis.

[31] Kleinbaum D.G., Klein M., Kleinbaum D.G., Klein M. (2010). Ordinal Logistic Regression, in Logistic Regression: A Self-Learning Text.

[32] StataCorp (2017). Stata Statistical Software.

[33] Rohrmann S., Linseisen J. (2016). Processed meat: The real villain?. Proc. Nutr. Soc..

[34] Boada L.D., Henríquez-Hernández L., Luzardo O. (2016). The impact of red and processed meat consumption on cancer and other health outcomes: Epidemiological evidences. Food Chem. Toxicol..

[35] Packaged Facts (2017). The Meal Kit Delivery Services Market. https://www.packagedfacts.com/Content/Featured-Markets/Meal-Kit-Delivery-Services.

[36] Acciai F., Belarmino E., Josephson A., Niles M. (2020). Changes in Employment Status and Food Security during the COVID-19 Pandemic.

[37] Jones J. (2021). Online Supplemental Nutrition Assistance Program (SNAP) Purchasing Grew Substantially in 2020, in Amber Waves. https://www.ers.usda.gov/amber-waves/2021/july/online-supplemental-nutrition-assistance-program-snap-purchasing-grew-substantially-in-2020/.

[38] USDA Food and Nutrition Service (2022). Stores Accepting SNAP Online. https://www.fns.usda.gov/snap/online-purchasing-pilot.

[39] Kuroko S., Black K., Chryssidis T., Finigan R., Hann C., Haszard J., Jackson R., Mahn K., Robinson C., Thomson C. (2020). Create Our Own Kai: A Randomised Control Trial of a Cooking Intervention with Group Interview Insights into Adolescent Cooking Behaviours. Nutrients.

[40] Pope L., Alpaugh M., Trubek A., Skelly J., Harvey J. (2021). Beyond Ramen: Investigating Methods to Improve Food Agency among College Students. Nutrients.

[41] Robinson J., Bertmann F., Harper K., Biehl E., Neff R. (2020). US Consumer Experiences with Food Access During Covid-19. http://jhir.library.jhu.edu/handle/1774.2/63253.

[42] Brandt E.J., Silvestri D.M., Mande J.R., Holland M.L., Ross J.S. (2019). Availability of Grocery Delivery to Food Deserts in States Participating in the Online Purchase Pilot. JAMA Netw. Open.

[43] Centers for Disease Control and Prevention (2022). COVID Data Tracker. https://covid.cdc.gov/covid-data-tracker.

